# Saving lives: assessing knowledge and attitudes toward basic life support among teachers and parents in Tabuk City

**DOI:** 10.3389/fmed.2025.1609761

**Published:** 2025-07-08

**Authors:** Amirah M. Alatawi

**Affiliations:** Department of Community and Family Medicine, Faculty of Medicine, University of Tabuk, Tabuk, Saudi Arabia

**Keywords:** basic life support, teachers, parents, knowledge, attitude, cardiopulmonary resuscitation, Saudi Arabia

## Abstract

**Background:**

Assessing the current awareness levels of teachers and parents about basic life support is essential for identifying training gaps, obstacles to acquiring basic life support skills, and areas that require improvement.

**Aim:**

This study aimed to evaluate the knowledge, skills, and attitudes concerning basic life support among kindergarten and primary school teachers and parents in Tabuk City, Saudi Arabia.

**Methods:**

This cross-sectional survey was conducted in Tabuk City, Saudi Arabia, involving teachers and parents of children enrolled in kindergartens or elementary schools, aged 18 years or older. Data were collected using a previously validated, self-administered Arabic questionnaire, distributed through an online link via social media Apps. The total knowledge and skills score was then calculated.

**Results:**

Most participants (70.3%) were parents, with ages ranging from 18 to 70 years and an average age of 35.1 ± 10.91 years. Female participants (59.7%) outnumbered males. Over half (53.3%) had no prior cardiopulmonary resuscitation (CPR) training, and 55.6% had never witnessed CPR being performed. Approximately half (50.3%) demonstrated a fair level of knowledge, while 44.6% had poor levels, and only 5.1% achieved a good level. A significant association was found between previous CPR training and knowledge and skill levels (*p* < 0.001). Additionally, the majority (81.0%) expressed interest in receiving further CPR training.

**Conclusion:**

This study identified substantial gaps in basic life support awareness and training among kindergarten and primary school teachers and parents in Tabuk City, Saudi Arabia. Most participants exhibited either poor or average levels of knowledge, with critical deficiencies in CPR skills. Prior exposure to CPR training was significantly associated with higher knowledge and skill scores, underscoring the importance of accessible and structured training programs. The main barrier to training was a lack of awareness of available CPR courses. Nevertheless, the high level of expressed interest in future training highlights a strong potential for improving BLS competency through targeted educational initiatives.

## Introduction

Numerous child-related medical emergencies requiring instant intervention can occur at schools or homes (1). In such circumstances, teachers and parents share the responsibility of ensuring the safety and well-being of young children as they are frequently the first responders (2). Nevertheless, many teachers and parents lack the necessary training to respond effectively to emergencies, potentially increasing risks for children in critical situations (3, 4).

Basic life support (BLS) is provided by the first responders to assist individuals experiencing cardiac or respiratory arrest, and therefore it is crucial for preserving a child’s life. The emergency procedures provided in BLS include calling local emergency medical services, confirming cardiac arrest, and initiating cardiopulmonary resuscitation (CPR), besides the use of an automated external defibrillator (AED) (5).

It is imperative that non-health professionals, especially school guardians, receive BLS training to ensure the safety of the children. Training more people in CPR is a key part of strategies that improve community response to out-of-hospital cardiac arrests (OHCA). In this context, It has been reported that effective CPR and defibrillation can save half of OHCA (6).

Numerous studies from Greece (7), Belgium (8), Spain (9), and Columbus (3) have found that knowledge levels of BLS among school teachers and community members are inadequate. In addition, studies from Saudi Arabia involving school teachers and public individuals residing in Al-Qassim (10), Al-Khobar (11), Riyadh (12), and Taif (13) cities also documented unsatisfactory levels of knowledge regarding first aid and BLS.

Given the absence of prior studies on this issue in Tabuk City, it is essential to assess the current levels of awareness among teachers and parents regarding BLS. This step is essential to identify gaps in training and barriers to acquiring BLS skills as well as areas for improvement. Subsequently, a structured professional development program for teachers and community awareness initiatives for parents can be established. Therefore, this study aimed to evaluate the knowledge, skills, and attitudes concerning BLS among kindergarten and primary school teachers and parents in Tabuk City, Saudi Arabia.

## Methods

### Ethical considerations

The study received ethical approval from the Research Ethics Committee at the University of Tabuk, Saudi Arabia. Participants were invited to take part in the research and complete the questionnaire after providing their consent. At the beginning of the survey, the study objectives, methodology, risks, and benefits were explained. To maintain the confidentiality of data and ensure anonymity, each participant was given a unique code number.

### Study design, setting, and date

This cross-sectional survey study was carried out in Tabuk City, Saudi Arabia, from November 2024 to January 2025.

### Sample size and sampling technique

An online Raosoft sample size calculator “http://www.raosoft.com/samplesize.html” was used to calculate the valid sample size. Considering a 5% margin of error, a confidence interval of 95%, assuming an average response for most of the questions of 50%, and an estimated prevalence of adequate BLS knowledge of 50%, the minimum required sample size was 384 subjects.

A non-probability convenience sampling method was adopted to enroll the participants.

### Eligibility criteria

This study included teachers and parents of children attending kindergartens or elementary schools in Tabuk City who were aged 18 years or older, had access to the Internet, and agreed to participate.

Healthcare professionals with advanced BLS certification, respondents residing outside Tabuk City, and those who declined to provide consent or submitted surveys with incomplete data were excluded.

### Data collection procedure

After obtaining the ethical approval, data were collected using an online, self-administered questionnaire. The electronic survey was created using Google Forms (https://forms.gle/BY7WBurLgA2GRq2m9), and a link to the survey was disseminated via social media platforms such as “Facebook,” “Twitter,” “Instagram” and “WhatsApp.”

### Data collection instrument

A previously validated, self-administered Arabic questionnaire adopted from previous studies (13, 14) was used to collect data. The questionnaire was divided into five sections. The first section gathered the socio-demographic data of the participants. The second section focused on previous CPR training status and included questions about the place and time of training. The third section evaluated the participants’ knowledge and skills in performing BLS. The fourth section showed the attitudes toward training and practicing CPR, where the questions emphasized exploring their willingness and motivation to undergo training. The final section checked the participants’ experience in performing CPR.

The knowledge and skills were assessed by 10 questions, with 1 point for each correct answer and 0 points for each wrong answer. The overall knowledge and skills score was calculated by summing the scores for each item. The possible range of the total score was from 0 to 10 points. Finally, the total knowledge and skills score was categorized as good (8–10 points, 75% correct answers), fair (5–7 points, 50–74.9% correct answers), or poor (0–4 points, <50% correct answers). Regarding the participants’ attitudes, responses with “yes” were deduced as a positive attitude.

### Statistical analysis

All data were tabulated and analyzed by the statistical package for the social sciences software program, IBM SPSS Statistics for Windows, version 27 (IBM Corp., Armonk, NY, United States). We presented categorical data as frequencies and percentages and analyzed the possible associations between categorical variables using Pearson’s Chi-Square test. Description of the numerical data was done after checking for normality with the Shapiro–Wilk test and was presented as a mean and standard deviation. A cutoff of *p*-value less than 0.05 was considered statistically significant.

## Results

Table 1 demonstrates that most participants (70.3%) were parents, while 29.7% were teachers. The participants’ ages ranged from 18 to 70 years, with a mean age of 35.1 ± 10.91 years, and female participants (59.7%) outnumbered males. Most participants were Saudi citizens (95.1%). A great proportion of the participants were married (72.1%), while 23.8% were single and a few were divorced or widowed (2.1% each). More than half of the participants (54.6%) were graduates, and a smaller proportion had secondary (19.5%), middle (7.2%), or primary (2.8%) education. The educational field (37.9%) was the main employment sector, followed by non-employed participants (26.4%), and those in other fields (24.1%).

**Table 1:** Socio-demographic characteristics of the study participants (*N* = 390).

Variable	Category	*N* = 390	%
Age, years	Range	18.0–70.0	
	Mean ± SD	35.1 ± 10.91	
Respondent	Teacher	116	29.7%
	Parent	274	70.3%
Gender	Female	233	59.7%
	Male	157	40.3%
Nationality	Saudi	371	95.1%
	Non-Saudi	19	4.9%
Marital status	Married	281	72.1%
	Single	93	23.8%
	Divorced	8	2.1%
	Widowed	8	2.1%
Education level	Primary	11	2.8%
	Secondary	76	19.5%
	Middle	28	7.2%
	Graduate	213	54.6%
	Post-graduate	62	15.9%
Job sector	Educational field	148	37.9%
	Health field	45	11.5%
	Not employed	103	26.4%
	Others	94	24.1%

*N*, number; SD, standard deviation.

Regarding CPR training status, more than half (53.3%) of the participants lack previous CPR training. Among those who received training (*N* = 182), the place of training was either the schools (37.4%), the Red Cross (33.5%), or a private organization (16.5%). Strikingly, nearly half (46.7%) had received training more than 2 years ago, while 61 (33.5%) within the past 6 months. Queries about resuscitation experiences revealed that direct exposure to real-life resuscitation is relatively limited among participants where 55.6% had never observed CPR being performed, and only 14.4% of participants had actively participated in performing CPR (Table 2).

**Table 2:** Training background and resuscitation experiences of the study participants.

Item	Category	*N* = 390	%
Training status			
Have you received previous CPR training?	No	208	53.3%
	Yes	182	46.7%
Where and how have you received your CPR training? (*N* = 182)	In the school	68	37.4%
	Red cross	61	33.5%
	Private organization	30	16.5%
	Others	23	12.6%
Time since previous CPR training (*N* = 182)	0–6 months	61	33.5%
	7–12 months	22	12.1%
	13–24 months	14	7.7%
	More than 2 years	85	46.7%
Resuscitation experiences			
Have you observed CPR on a collapsed patient?	No	217	55.6%
	Yes	173	44.4%
Have you participated in CPR before?	No	334	85.6%
	Yes	56	14.4%

CPR, cardiopulmonary resuscitation.

Table 3 shows the responses to the knowledge and skills questions. The majority of participants (71.8%) correctly identified 997 as the emergency contact number in Saudi Arabia. When asked about the response to finding a lifeless adult, only 57.9% correctly reported the initial need to check for consciousness, secure the airway, and assess breathing before starting chest compressions. About two-thirds of participants (64.9%) correctly knew that a breathing but unresponsive person should be placed in the recovery position and contact the emergency services. When asked about the correct CPR technique, the correct placement of hands one above the other with palms downward was identified by 70.5, and 68.5% correctly answered that one must kneel next to the torso. Furthermore, 52.8% identified the correct ratio of 30:2 (30 chest compressions to 2 rescue breaths), while only 18.7% correctly answered that compressions should be at least 5 cm deep and at a rate of 100–120 compressions per minute, and only 16.4% properly recognized the recommended frequency of two compressions per second. When asked about AED use, only 44.1% had the correct knowledge that the purpose of AED is to analyze the heart rhythm and deliver an electric shock if necessary. However, only 6.9% correctly answered that any citizen is allowed to use an AED.

**Table 3:** Participants’ knowledge and skills in basic life support (*N* = 390).

Question	Response Option	*N* = 390	%
What is the correct emergency phone number?	997*	280	71.8%
	988	39	10.0%
	999	36	9.2%
	977	35	9.0%
You are alone and come across a lifeless adult person. What do you do?	Check for consciousness, secure airways, and check if the patient is breathing*	226	57.9%
	Check for pulse	127	32.6%
	Immediately start chest compressions	37	9.5%
It turns out the patient is breathing but shows no response to verbal stimuli. What do you do?	Put the patient in a recovery position and call for an ambulance*	253	64.9%
	Check for pulse	73	18.7%
	Immediately start chest compressions	64	16.4%
You decide to perform CPR. Which of the following combinations of chest compressions and ventilations would you choose?	30 chest compressions: 2 rescue breathings*	206	52.8%
	30 chest compressions: 5 rescue breathings	108	27.7%
	2 rescue breathings: 30 chest compressions	76	19.5%
How deep and how fast would you perform chest compressions?	I do not know	215	55.1%
	4–5 cm and 100/min	102	26.2%
	At least 5 cm and 100–120/min*	73	18.7%
What is the purpose of an AED?	To analyze the heart rhythm and give an electric shock if necessary*	172	44.1%
	I do not know	115	29.5%
	To give cardiac massage	67	17.2%
	To analyze the heart rhythm	36	9.2%
Who is allowed to use an AED?	Only skilled people	215	55.1%
	Only emergency personnel	148	37.9%
	Every citizen*	27	6.9%
Does the respondent kneel next to the torso?	Yes*	267	68.5%
	No	123	31.5%
How is the hand placement on the torso?	Place one hand above the other with the palms downward*	275	70.5%
	Cross the fingers of both hands	36	9.2%
	Use one hand as a fist	31	7.9%
	Use one hand	25	6.4%
	Two hands next to each other	23	5.9%
Chest compression frequency	One press/2 s	119	30.5%
	One press/1 s	71	18.2%
	Two press/1 s*	64	16.4%
	Less than one press/1 s	61	15.6%
	More than two presses/1 s	46	11.8%
	Irregular press	29	7.4%

AED, automated external defibrillator; *Refers to the correct answer for each question.

**Figure 1:**
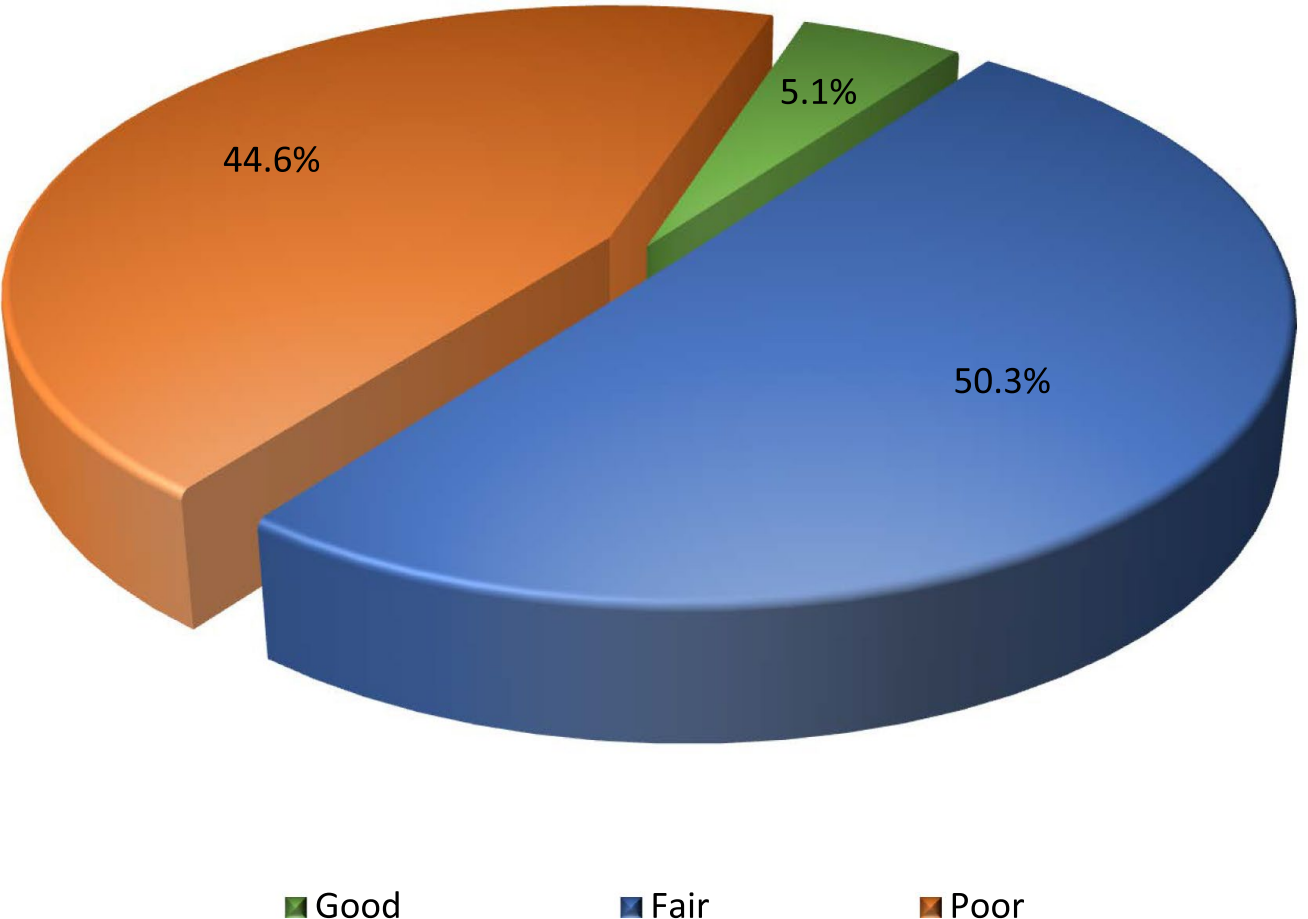
Levels of knowledge and skills in basic life support.

Figure 1 illustrates the distribution of participants’ knowledge and skill levels in BLS. About half (50.3%) showed a fair level, while poor levels constituted 44.6% and only a few participants (5.1%) exhibited good levels.

The analysis of the relationships between knowledge and skill levels in BLS and the participants’ sociodemographic characteristics including age, gender, nationality, education level, and job sector revealed the absence of statistically significant associations (All *p* values >0.05). Alternatively, previous CPR training showed a significant association with knowledge and skill levels (*p* < 0.001). Most (80%) participants classified as having good BLS knowledge and skills reported having prior CPR training, while 69.0% of those with poor knowledge and skills had never received CPR training as shown in Table 4.

**Table 4:** Associations between knowledge and skills levels and the participants’ sociodemographic and training characteristics.

Sociodemographic and training characteristics		Knowledge and skills level						*P*-value
		Good *N* = 20 (5.1%)		Fair *N* = 196 (50.3%)		Poor *N* = 174 (44.6%)		
Respondent type	Teacher	6	30.0%	55	28.1%	55	31.6%	0.757
	Parent	14	70.0%	141	71.9%	119	68.4%	
Age group, years	<25	3	15.0%	30	15.3%	29	16.7%	0.503
	25–35	6	30.0%	89	45.4%	69	39.7%	
	36–45	4	20.0%	39	19.9%	44	25.3%	
	46–55	5	25.0%	32	16.3%	28	16.1%	
	>55	2	10.0%	6	3.1%	4	2.3%	
Gender	Female	12	60.0%	111	56.6%	110	63.2%	0.435
	Male	8	40.0%	85	43.4%	64	36.8%	
Nationality	Saudi	20	100.0%	186	94.9%	165	94.8%	0.583
	Non-Saudi	0	0.0%	10	5.1%	9	5.2%	
Educational level	Primary	0	0.0%	6	3.1%	5	2.9%	0.725
	Secondary	4	20.0%	43	21.9%	29	16.7%	
	Middle	1	5.0%	15	7.7%	12	6.9%	
	Graduate	11	55.0%	108	55.1%	94	54.0%	
	Post-graduate	4	20.0%	24	12.2%	34	19.5%	
Job sector	Educational field	6	30.0%	72	36.7%	70	40.2%	0.267
	Health field	5	25.0%	27	13.8%	13	7.5%	
	Not employed	5	25.0%	52	26.5%	46	26.4%	
	Others	4	20.0%	45	23.0%	45	25.9%	
Previous CPR training	No	4	20.0%	84	42.9%	120	69.0%	<0.001*
	Yes	16	80.0%	112	57.1%	54	31.0%	
Time since received CPR training (*N* = 182)	0–6 months	7	43.8%	35	31.3%	19	35.2%	0.536
	7–12 months	3	18.8%	12	10.7%	7	13.0%	
	13–24 months	2	12.5%	9	8.0%	3	5.6%	
	More than 2 years	4	25.0%	56	50.0%	25	46.3%	

CPR, cardiopulmonary resuscitation; *Significant at *p* < 0.05.

Table 5 provides intuition into the participants’ barriers and attitudes toward CPR training and BLS application. The participants who had never received CPR training reported different reasons such as not knowing where to attend a course (27.7%), lack of time (22.6%) or interest (14.1%), or financial costs (5.9%). Asking about the concerns that prevent the participants from performing BLS revealed fear of causing harm to the victim (37.2%) or lack of knowledge and skills (34.1%), besides infection risks (12.8%) and legal concerns (9.0%). Most (81.0%) participants expressed interest in receiving more CPR training, and this frequency was increased to 85.4% if a free CPR course was offered. The main motivation for CPR training was to prevent unnecessary deaths (35.1%). High percentages supported mandatory CPR training either in schools (40.5%), or for all jobs (37.9%). Furthermore, 59.5% supported requiring CPR training for teacher certification, while 40.5% showed a divergent response. More than half (57.2%) believed every school should have an AED, while 42.8% disagreed.

**Table 5:** Perceptions of the study participants toward cardiopulmonary resuscitation training.

Question	Response Option	*N* = 390	%
If you had no previous CPR training, what is the reason?	Costs	23	5.9%
	Lack of interest	55	14.1%
	Lack of time	88	22.6%
	Not sure where to attend course	108	27.7%
	No answer	116	29.7%
What reason makes people afraid to apply BLS to victims?	Causing potential harm to the person in need	145	37.2%
	Afraid of contagious diseases through mouth-to-mouth breath	50	12.8%
	Afraid of legal consequences	35	9.0%
	Emotional reasons	27	6.9%
	Lack of proper knowledge and skills	133	34.1%
Do you want more training?	No	74	19.0%
	Yes	316	81.0%
If you want more CPR training, what is the reason?	Heart disease within the family	29	7.4%
	Wish of avoiding unnecessary death	137	35.1%
	Others	119	30.5%
	No answer	105	26.9%
Would you be willing to take a free CPR course if it were offered?	No	57	14.6%
	Yes	333	85.4%
Do you think CPR training should be mandatory?	No, CPR training should be optional	56	14.4%
	Yes, at school	158	40.5%
	Yes, to obtain a driver’s license	28	7.2%
	Yes, training should be mandatory in every job	148	37.9%
Is CPR already part of the educational curriculum?	No	214	54.9%
	Yes	176	45.1%
Do you think CPR training should be required for teacher certification?	No	158	40.5%
	Yes	232	59.5%
Do you think each school should have an AED?	No	167	42.8%
	Yes	223	57.2%

AED, automated external defibrillator; CPR, cardiopulmonary resuscitation.

## Discussion

This study revealed critical gaps in awareness and training in BLS among kindergarten and primary school teachers and parents in Tabuk City, Saudi Arabia. The majority of participants had either an average level of awareness (50.3%) or lacked the adequate levels necessary for emergencies (44.6%). Only a very small percentage (5.1%) demonstrated a good level. Specific gaps in essential CPR details were identified including the correct compression-to-breath ratio, compression depth and frequency, and the purpose and the person who can use the AED. These findings suggest insufficient or inaccessible current training programs. Moreover, the fact that half (50.3%) of the participants exhibited a “fair” level of knowledge denotes that the current knowledge level may not be strong enough to ensure confident and effective intervention in emergencies. Exploration of the current training status of the participants revealed limited training opportunities where more than half of (53.3%) the participants have never received any formal CPR training, and nearly half (46.7%) of the trained participants had received their CPR training more than 2 years ago. Up-to-date training within the past 6 months was reported by only 33.5% of the respondents. Additionally, the current survey documented limited real-life exposure and hands-on experience in CPR. A substantial number of participants (55.6%) had never observed CPR being performed, and only 14.4% had actively participated in performing CPR.

In agreement with these findings, teachers working in Al-Madinah City (14) and Al-Qassim Region (10), Saudi Arabia exhibited comparable inadequate knowledge of BLS, particularly low knowledge regarding CPR skills and AED use. The majority of teachers also reported having no previous CPR training and most of them had training more than 2 years ago. Another study in Taif City involved 248 teachers and 400 parents established poor knowledge levels about CPR skills among more than 75% of the respondents, with no significant difference between teachers and parents (13). Likewise, studies from Riyadh (12) and Buraidah (15), Saudi Arabia explored the limited availability of CPR courses for school teachers as well as mothers. Internationally, earlier studies from the United Kingdom and the United States also showed low knowledge levels about CPR among teachers (16–18).

The present study also found that BLS knowledge and skill levels are primarily determined by training rather than demographic factors. This underlines the importance of increasing access to CPR training programs to improve overall awareness and skills in BLS. Regular refresher courses are essential as they affect the retention of knowledge and the ability to perform CPR effectively. Organizations and policymakers should prioritize structured CPR training, as well as refresher courses, to ensure a greater number of individuals achieve satisfactory responses in emergencies. Various studies documented a significant association between previous CPR training courses and knowledge levels (2, 19, 20). The necessity of regular CPR training to improve knowledge and skills has been also reported by Nori et al. (21) who also suggested the implementation of BLS courses in the educational curriculum. Furthermore, López et al. (9) and Khademian et al. (22) conducted interventional studies and concluded a positive impact of BLS training on the knowledge and skills of teachers and public individuals with improvement in CPR sequence and quality of chest compression after training. The detected deficiency in knowledge about the AED use in this study should be considered while preparing for the training programs as both high-quality CPR and rapid defibrillation are essential for good outcomes of out-of-hospital cardiac arrest (23, 24).

The present study investigated the key barriers preventing participants from receiving CPR training and performing BLS. Participants stated that they did not attend CPR training because they had no information about the places of the CPR courses. The same reason was reported in previous Saudi studies (10, 14), and a study from Palestine (25). Alternatively, an earlier study from the United States highlighted the financial cost of CPR training as the main barrier (3). In addition, lack of knowledge and skills and fear of causing harm to the victim were the most common reasons for their hesitation in performing BLS measures. This emphasizes the necessity for increasing awareness and hands-on training to boost their confidence.

Most participants in this study showed positive attitudes and strong support for CPR training. Most of them want to learn CPR skills and the main motive for learning was to avoid unnecessary deaths. Similar findings were reported in the previous studies from different countries (8, 10, 14, 20). Many participants believed in the need for early education and supported making CPR training mandatory in schools, and to be as a requirement for teacher certification. Some suggested extending CPR training to be a prerequisite for all jobs. Despite more than half favoring the presence of AEDs in schools, there were some concerns probably due to the cost, maintenance, or usability. It is worth mentioning that many American states, Canada, and several European countries have legislated CPR training in schools to ensure that individuals acquire this crucial skill at a young age, increasing the likelihood that they will retain it into adulthood, enhancing confidence and reducing fear (26, 27). The implementation of AED programs in schools has shown a high survival rate for individuals experiencing sudden cardiac arrest in high schools across the United States (28). To effectively implement CPR training in schools, clear guidelines should be established on the required proficiency level for students, along with training programs for teachers. It is also essential to emphasize that advanced skills are not necessary to perform CPR. Additionally, familiarizing teachers with CPR training kits can help enhance the learning process. A multicenter study concluded that effective CPR training implementation in schools dictates establishing clear guidelines for the required proficiency level for both the students and the teachers. It also emphasized the importance of familiarizing teachers with CPR training kits (29).

## Limitations

The cross-sectional study design, besides the reliance on self-reported data, may introduce bias or inaccuracy into the results. In addition, the use of a non-random sampling method may limit the ability to generalize the results to the entire population.

## Conclusion

This study highlights substantial gaps in BLS awareness and training among kindergarten and primary school teachers and parents in Tabuk City, Saudi Arabia. Most participants demonstrated either average or inadequate levels of knowledge, with critical deficiencies in fundamental CPR skills, particularly concerning compression techniques and the effective utilization of AEDs. Training exposure emerged as the only factor influencing knowledge and skill levels. A lack of awareness regarding available CPR training opportunities was the most frequently cited barrier to participation. Despite these gaps, participants exhibited a predominantly positive attitude toward acquiring BLS skills, with a strong willingness to engage in further training. These findings underscore the urgent need to improve the awareness of teachers and the public about BLS by increasing access to structured CPR training programs and incorporating practical, hands-on components to foster confidence and preparedness in emergency situations.

## Data Availability

The original contributions presented in the study are included in the article/supplementary material, further inquiries can be directed to the corresponding author.
